# Musculoskeletal Disorders Symptoms among Taiwanese Bakery Workers

**DOI:** 10.3390/ijerph17082960

**Published:** 2020-04-24

**Authors:** Yi-Lang Chen, Yan-Ting Zhong, Bang-Nan Liou, Chih-Chuan Yang

**Affiliations:** 1 Department of Industrial Engineering and Management, Ming Chi University of Technology, New Taipei 24301, Taiwan; U04217151@o365.mcut.edu.tw (Y.-T.Z.); U04217141@o365.mcut.edu.tw (B.-N.L.); U04217135@o365.mcut.edu.tw (C.-C.Y.); 2 Department of Industrial Design, Chang Gung University, Touyuan 33302, Taiwan

**Keywords:** bakery work, musculoskeletal disorders, Nordic Musculoskeletal Questionnaire (NMQ), risk factors, wrist movements

## Abstract

In this study, the Nordic Musculoskeletal Questionnaire (NMQ) was administered to a valid sample of 81 Taiwanese bakery workers to explore their discomfort or symptoms of work-related musculoskeletal disorders and identify the risk factors. Wrist postures were also examined during 3 typical dough operations (kneading, rolling, and rounding) by using an electrogoniometer. The prevalence of musculoskeletal discomfort in any part of the body in the past year among the respondents was 93.0%, with the highest prevalence of 66.3% and 51.8% in the hands/wrists (right and left), followed by the prevalence of 50.6% and 45.8% in the shoulders (right and left) and the lower back (48.2%), respectively. The results also revealed that during the 3 dough processing operations, the workers’ wrist movements in specific operations were close to the recommended limits suggested in previous studies, especially the ulnar deviation and palm flexion of the right wrist during dough kneading and the radial deviation of the left wrist during dough rolling and rounding. The study findings can be used to explain why the bakers self-report a high proportion of wrist and shoulder disorders and can also serve as a reference for task rearrangement and redesign.

## 1. Introduction

Studies have described work-related musculoskeletal disorders (WMSDs) as endemic in a wide spectrum of occupational groups, including industrial workers, people involved in food and meat processing, and clerks [1,2,3]. WMSD is frequently caused by prolonged exposure to highly repetitive work, and its prevalence and the associated medical and indemnity costs have increased annually [4]. WMSDs in the upper limbs are common in people in various professions and industries worldwide, particularly frontline workers, due to awkward postures, overexertion, and repetitive work [5,6,7]. Among the WMSDs, carpal tunnel syndrome (CTS) is a common wrist injury among frontline workers and data processors [8,9,10,11].

Baking is a typical repetitive task involving the upper limbs, especially the wrists and shoulders. However, the research on the health conditions of bakery is lacking. Currently, Taiwan has more than 10,000 bakeries and specialty stores, not including convenience stores that also supply bakery products. Suppose that 5 people are employed at each store; then, we can estimate that there are approximately 50,000 employees in the industry in Taiwan. Due to the small scale of most stores and cost considerations, the dough processing operation is still manual, which has long caused musculoskeletal disorders in employees. Most studies on the health of bakery workers have mostly focused on industrial bakeries in high-income countries, with emphasis on the effects of flour dust on the respiratory system [12,13,14]) or the allergies and skin problems it causes [14,15]. Forcier et al. compared musculoskeletal pain in workers among 7 departments of supermarkets in Canada and found that bakers were the second most likely to experience pain among all groups [13]. Habib and her colleagues [16] found that 23% of bakery workers reported upper extremity pain. Workers who reported poor health and those who perceived that the work environment negatively affects their health were twice as likely to experience upper extremity musculoskeletal pain. However, the healthy worker survivor effect should be considered; it means those that we found at bakeries are the survivors.

**Figure 1:**
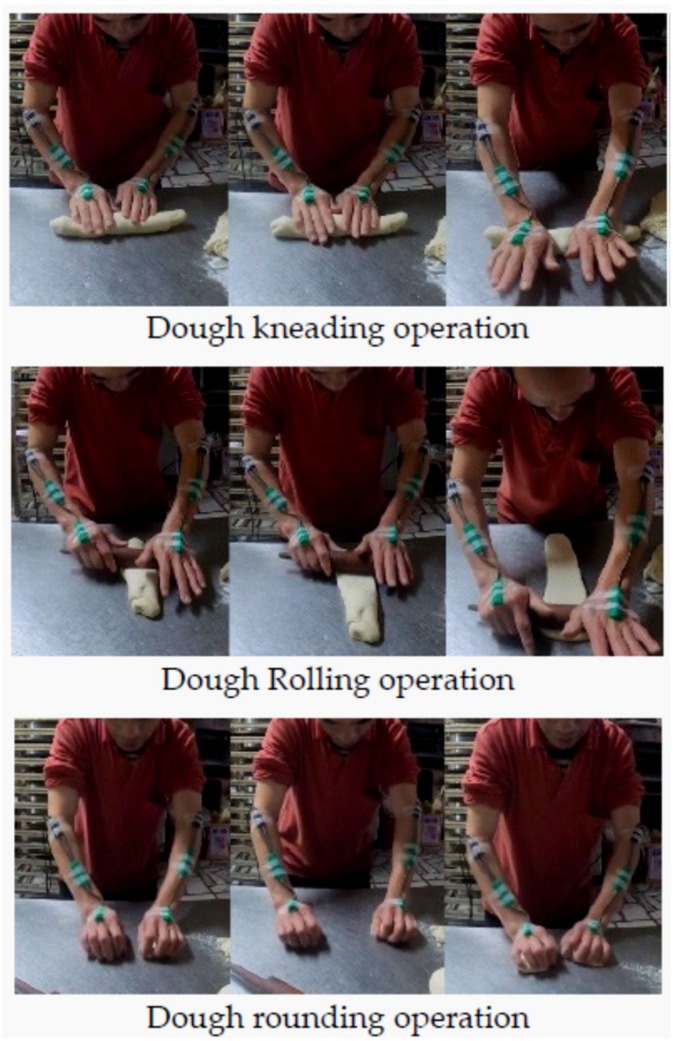
Three types of tasks usually performed by bakery workers.

Our pilot study results revealed that dough processing by using hands is one of the primary daily tasks of bakery workers. Many consumers still believe that hand-kneaded bread is particularly delicious. Dough processing typically involves 3 operations: kneading, rolling, and rounding, as shown in Figure 1. Since dough processing involves many procedures and the division of labor in each store is different, not all bakery workers are engaged in these 3 dough operations, which may explain the reason for different WMSDs prevalence among bakery workers. Bakery workers are regularly exposed to strenuous manual activities including heavy lifting, forceful exertions, and awkward postures [16]. Several workplace conditions and tasks performed by workers might contribute to symptoms of WMSD, including dough handling, standing for prolonged periods next to a hot oven, continuous bending to insert heavy trays in the oven, lifting and moving of heavy items and bags sometimes up and down a staircase, and working in cramped and heated spaces. Bakery workers also work for long hours, in night and early morning shifts, and they are under pressure to complete certain tasks in a limited duration [16]. These potentially harmful work practices in bakeries may lead to musculoskeletal problems among workers. Therefore, investigating the association between musculoskeletal disorders and task characteristics in nonindustrialized bakeries is necessary to determine possible task redesign and improvement solutions.

## 2. Materials and Methods

In this study, we used in-depth interviews to modify the Nordic Musculoskeletal Questionnaire (NMQ) and conducted a survey of musculoskeletal disorders among Taiwanese bakery workers. Then, logistic regression was applied to determine the potential risk factors for the symptoms. Furthermore, 3 manual dough processing operations were monitored using goniometers to examine the wrist movement during the operations and assess whether the wrists were subjected to awkward postures.

### 2.1. NMQ

A convenience sample of 87 full-time employees with at least 1 year of work experience was recruited into the study. The NMQ was administered one-on-one to each of 87 employees from 23 bakeries in Northern Taiwan to collect data on their work characteristics, which were based on and revised from the previous questionnaire developed and adapted by the Institute of Labor and Occupational Safety and Health (ILOSH), Taiwan [17]. Data from 81 employees were finally analyzed. The questionnaire was distributed between 1 January and 15 March 2019. The NMQ is a general questionnaire that classifies musculoskeletal discomfort or symptoms of the disorders according to 9 major sites, and it enables researchers to discriminate among sites of discomfort and injury and closely examine symptoms specific to certain sites. A special map facilitating the identification of any particular body part was used [18]. The NMQ can be either general for all body or specific for each body part. Takekawa et al. concluded that the NMQ is the most effective instrument for identifying respondents with chronic or recurring low back pain [19]. Deakin et al. also found that this self-report questionnaire has reliability ranging from 77% to 100% and validity ranging from 80% to 100% [20]. The questionnaire is also suitable for use in the Taiwanese population [21,22]. In this study, informed consent was obtained from all participants, and the Ethics Committee of Chang Gung Memorial Hospital approved the study protocol.

In addition to a general descriptive statistical analysis, a logistic regression analysis was conducted to explore the possible risk factors for the symptom or discomfort at each site [23]. The following data were collected using the questionnaire: (a) personal details (including age, weight, height, dominant hand, work experience, exercise, tobacco smoking, alcohol consumption, and health and medical backgrounds) and (b) job demands (including number of working days per week, number of working hours per day, number of times the worker needs to perform the task of removing pastries out of the oven per day, and frequencies of rolling pin use, dough kneading task, dough rounding task, and object lifting).

### 2.2. Measurement of Wrist Movement

Three typical bakery workers (men aged 38–52 years with at least 5 years of work experience in a bakery) were recruited from a pastry shop in New Taipei City. They had no history of musculoskeletal disorders and were requested to perform 3 specific daily bakery operations (i.e., roll out the dough, knead the dough, and round the dough; Figure 1). We used a twin-axis electrogoniometer (SG 150, Biometrics, UK) to record alterations in wrist postures, including palmar flexion, dorsiflexion, ulnar deviation, and radial deviation, for each participant during the bakery operations. The wrist movement data during the 3 operations were recorded for a sampling period of 20 s in each trial, and the sample frequency was set at 16 Hz. Figure 1 also shows the goniometer used in this study, which contains 2 bases connected to the signal collection device worn by the participants. The two bases of the goniometer were attached on the dorsal surface using double-sided tape: one end over the third metacarpal and the other over the midline of the forearm. After the bases were attached, the electric potential for neutral wrist postures measured by the goniometer was documented, and the calibration base was set. Once the wrist deviates from this neutral posture, it means the movement occurrence. The electrogoniometer device has two separate output connectors: one is measuring palmar flexion/ dorsiflexion, and the other is measuring radial/ulnar deviation. After becoming familiar with the experiment procedure, each participant performed the 3 bakery operations. The participants were allowed a 5-min break between each trial and were instructed to work with the dough using their usual method and pace.

Data were collected using the electrogoniometer for 3 cycles continuously, and one of the cycles was randomly selected, and the corresponding data were further analyzed for each operation. Each operation lasted approximately 20 s. After the data were rearranged in order, the 95th percentile values for any wrist position deviating from the neutral position in each trial were used for further analysis, because these positions exhibited relatively extreme movement and may cause discomfort at the wrist and even injury.

### 2.3. Statistical Analysis

Statistical analyses were conducted using SPSS 22.0 (IBM Corp., Armork, NY, USA), with significance defined as a minimum α level of 0.05. Questionnaire data were examined through descriptive statistics and logistic regression. The odds ratio (OR) was used to compare the relative odds of the occurrence of different variables, such as personal details and job demands.

## 3. Results

### 3.1. Demographics

A total of 87 respondents were surveyed in this case study, and 81 valid questionnaires were recovered. The effective recovery rate was 93.1%. Of the 81 respondents, 70 were men and 11 were women; the average (standard deviation, SD) age was 31.0 (11.8) years, with a wide range from 20 to 65 years; the average height and weight were 171.0 (7.9) cm and 67.9 (14.0) kg, respectively. Among the respondents, 35.0% did not exercise, 43.0% exercised occasionally (1 or 2 times a week), and only 22.0% exercised regularly (more than 3 times a week). Among all respondents, 20.0% and 63.0% had the habit of smoking and drinking, respectively. The left and right dominant hands were 12% and 88%, respectively. In addition, 39.5% of the respondents reported that before engaging in bakery work, they had pain or discomfort in their body parts, namely wrist injuries (11.1%), shoulder pain (21.0%), upper back pain (13.6%), and lower back pain (19.8%); nearly one-fourth of the respondents still reported pain at the time of the interview.

### 3.2. Work-Related Characteristics

The average (SD) job tenure of 81 respondents was 8.8 (10.9) years; the sleep time and daily worktime (excluding resting time) were 6.5 (0.9) and 7.9 (2.2) hours, respectively, of which the total daily resting time was approximately 35.8 min; and the standing time during working hours was 6.5 (1.9) hours. Of the interviewees, 80% had been trained professionally, 33.7% needed to make more than 100 pieces of dough per day, and nearly 70% took the time to engage in the dough operation and it occupied more than half of their working time. In summary, the normal daily bakery tasks were characterized by prolonged standing times and extremely high dough operating times.

### 3.3. NMQ Results

Among the 81 bakers, the overall prevalence of disorders in any part of the body within a year was 93.0%, with the highest prevalence in the hands/wrists (66.3% (right) and 51.8% (left)), followed by shoulders (50.6% (right) and 45.8% (left)), lower back/waist (48.2%), neck and upper back (32.5%), and right (28.9%) and left elbows (19.3%), as shown in Table 1. A further analysis showed that the main disorder symptoms in the upper limbs of the respondents were soreness and pain. Most of the respondents indicated that the symptoms slightly reduced their ability to work, and the frequency of their occurrence was almost daily, but most of these symptoms were neglected or left untreated. The treatment for the symptoms was massage practices, and most believed that the disorder was due to the work.

This study further employed logistic regression to examine the risk factors for discomfort in the body parts of bakers. The 5 parts of the upper limbs with a high prevalence of discomfort (>45%) were individually analyzed, and the risk factors were identified. Table 2 presents the factors causing the disorders in the hands/wrists, shoulders, and lower and upper back. The results indicate that the dough operation and amount of exposure were the chief causes of musculoskeletal discomfort and significantly affected discomfort in the hands/wrists, shoulders, and lower back/waist.

### 3.4. Wrist Movements

**Figure 2:**
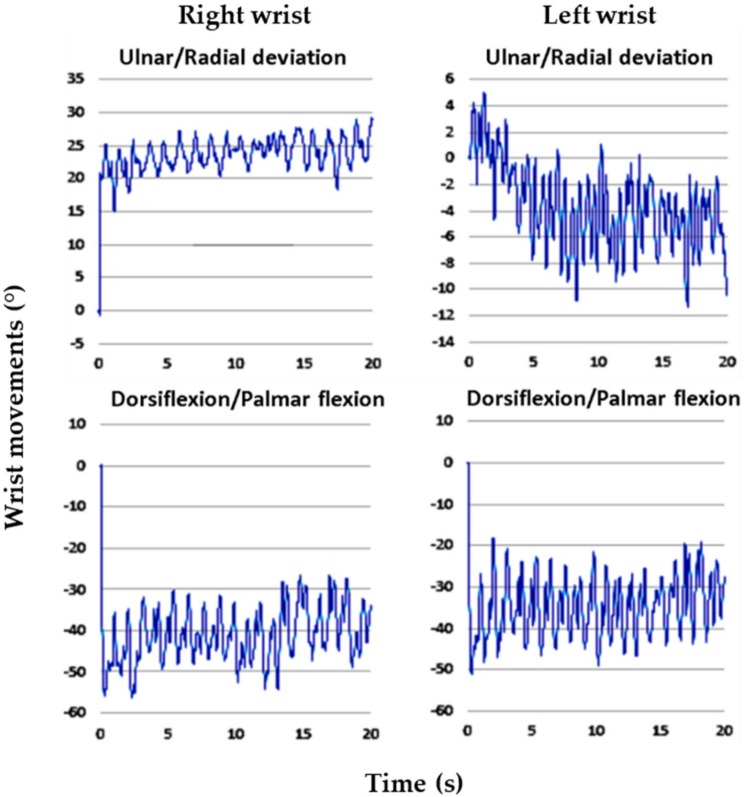
Alterations in the wrist posture during dough kneading.

**Figure 3:**
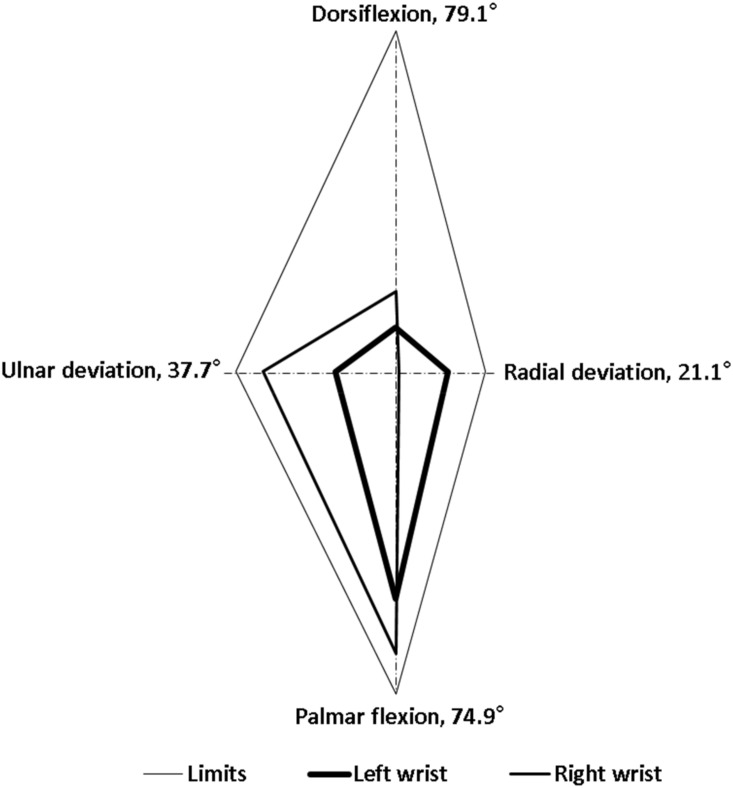
Maximum range of motion of the wrist joint during dough kneading.

**Figure 4:**
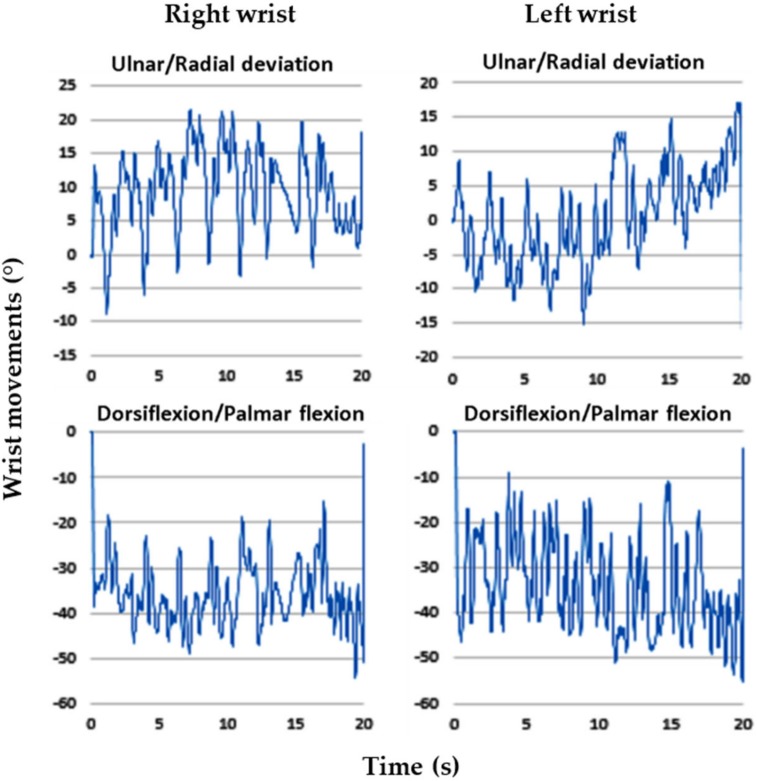
Alterations in wrist posture during the dough rod operation.

**Figure 5:**
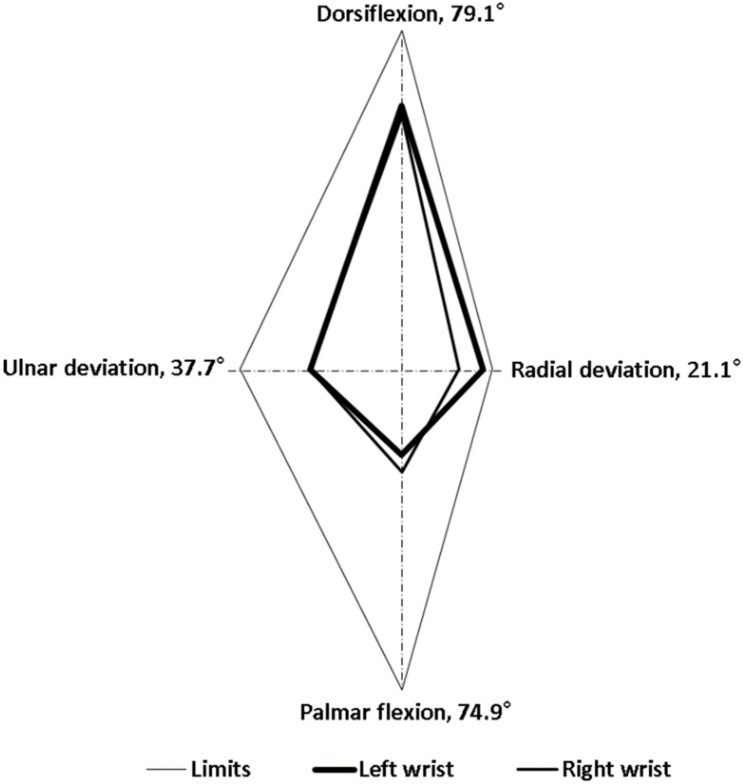
Maximum movement range of the wrist joint during the dough rod operation.

**Figure 6:**
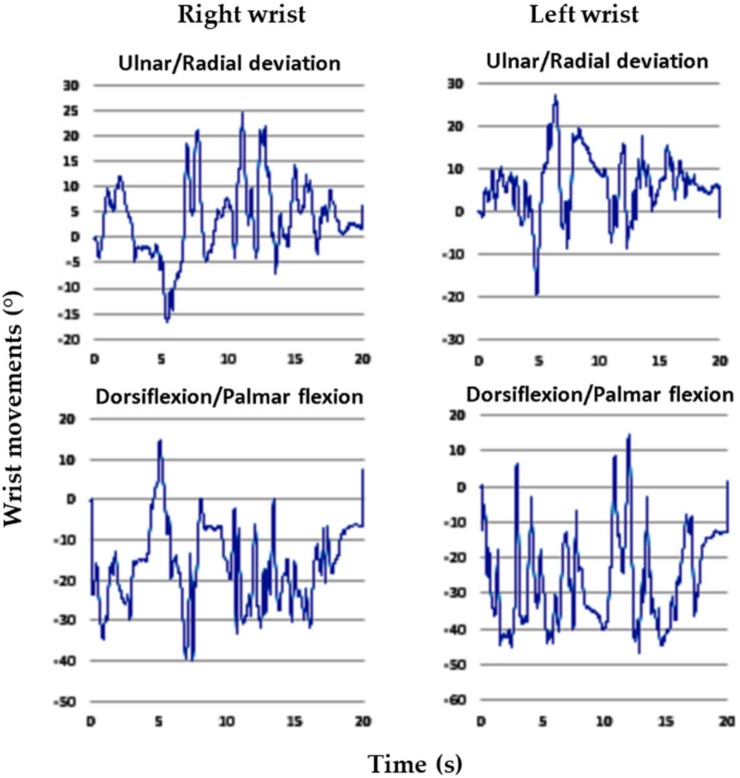
Wrist posture alterations during dough rounding.

**Figure 7:**
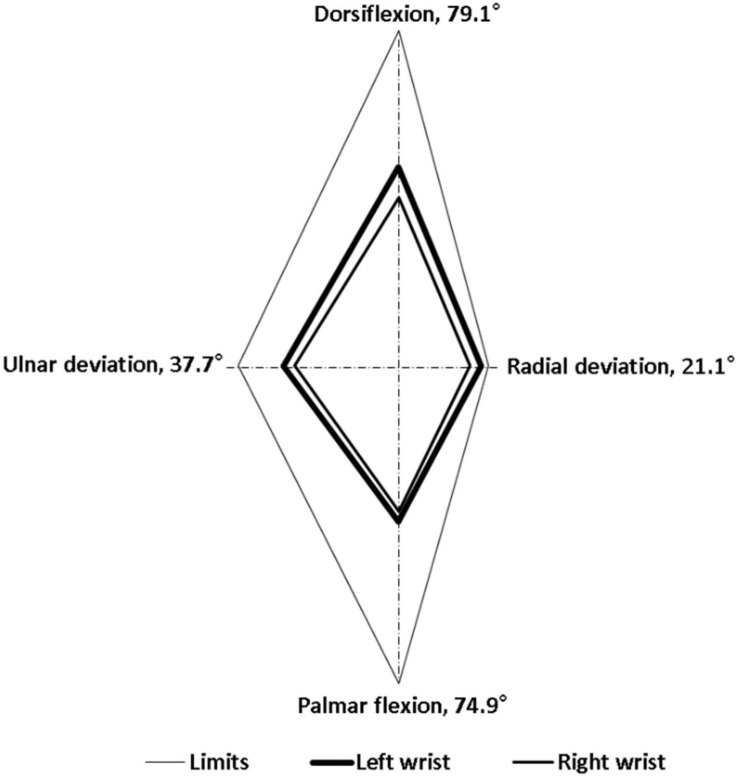
Maximum range of motion of the wrist joint during dough rounding.

Regarding goniometer measurements, alterations in the wrist postures of the 3 experienced employees were highly consistent. For the entire cycle of dough operations, the time spent by the employees was 20 s. The intraemployee variabilities in the right and left wrist maximum movements were less than 4.1° and 4.7°, respectively. This suggested that the employees were proficient in the dough operations. In this study, we selected the data of one representative employee for analysis because the wrist alterations were highly consistent among the employees. Figure 2, Figure 3, Figure 4, Figure 5, Figure 6 and Figure 7 show alterations in wrist palmar flexion, dorsiflexion, ulnar deviation, and radial deviation when performing 3 dough operations during a normal cycle and the maximum movement range of the wrist joint during the operations. The safety limits of human wrists suggested by Stetson et al. [24] were also highlighted in the figures. During the 3 types of operations, the maximum ulnar deviation/dorsiflexion of the right wrist in the workers was close to the movement limits defined in the literature, and the same phenomenon was observed for the left ulnar deviation.

## 4. Discussion

In the analyses, the overall prevalence of disorders in any part of the body within a year was 93.0% among the 81 bakers, with the highest prevalence in the hands/wrists (66.3% in right and 51.8% in left) and shoulders (50.6% in and 45.8% in left). The prevalence in the present study was higher than that in a survey by Habib et al., in which 23% of bakery workers reported upper extremity pain [16]. A comparison of the results of this study with the results of a newest survey conducted by the Institute of Labor and Occupational Safety and Health (ILOSH) in Taiwan [25] revealed that the prevalence of disorders in the bakers’ hands/wrists, shoulders, and lower back/waist was higher than that in workers of other industries (Table 1). This result may be related to the dough operations. Although the standing time was long (6.5 h per day), the prevalence of lower limb discomfort was not significant. For bakery workers, upper limb injuries may be the main concern. It should be noted that most of the respondents reported that the symptoms slightly reduced their ability to work, and the frequency of their occurrence was almost daily, but most of these symptoms were neglected or left untreated. This may be attributed to their socioeconomic status [26], because if they need to be at work to ensure financial support for their families, they neglect the WMSD symptoms and also the disorders, so they stop working only when it’s impossible to continue. This may cause them to fail to fully recuperate and make the symptoms worse.

Logistic regression results (Table 2) show that, relative to very little exposure (<10% within a workday), frequent rolling operation had a significant effect on all upper limb discomfort conditions, and the amount of dough kneaded could also cause hands/wrist discomfort. The factors causing discomfort in the shoulders and lower back included the need to lift objects frequently (such as moving flour bags) and the frequency of removing pastries out of the oven per day (>20 trays per day). In addition, working hours per day, job tenure, and age affected the prevalence of shoulder discomfort, whereas age also caused lower back discomfort. Notably, stature was also a significant factor affecting hand/wrist discomfort. The respondents whose stature was >170 cm exhibited a high degree of discomfort. This may be attributed to the potential influence of 11 female respondents in the survey. Women are usually not assigned for performing the dough operations in bakeries. Whether the relatively low-height working table was harmful to these taller workers requires further clarification.

Typically, when ulnar or radial deviation of the wrist and palmar flexion or dorsiflexion exceeds the normal range of motion (ROM), CTS may occur. Studies have demonstrated that the ROM of palmar flexion and dorsiflexion of the wrist is greater than that of ulnar and radial deviation [27,28]. Specifically, Ryu et al. [27] found that the largest ROM of human wrists is 59.3° for dorsiflexion, 79.1° for palmar flexion, 37.7° for ulnar deviation, and 21.1° for radial deviation. This finding is consistent with that in previous studies, with the exception of the ROM for dorsiflexion, which is lower than the value of 74.9° reported by Boone and Azen [29]. Stetson et al. also defined an involuntary wrist deviation when the wrist underwent flexed, extended, radial/ulnar deviations to 30°, 45°, and 18°, respectively [24]. These positions can be considered the safety limits of wrist movement.

As shown in Figure 2, the frequency of kneading was approximately 1.2 times/s, which is much higher than the safety limit of 0.1–0.5 times/s recommended by Lin and Radwin [30]. Figure 3 further indicates that the maximum dorsiflexion (65.5°) and ulnar deviation (31.3°) of the right wrist were close to the limit of movement suggested by Ryu et al. [27]. Compared with the right wrist, the dorsiflexion of the left wrist was relatively small, but it still exceeded the acceptable range. During dough kneading, the wrist was subjected to an unsafe posture, thus causing discomfort and even injury of the wrists. During the dough rolling operation, wrist activity was similar to that observed during the kneading operation, with a highly repetitive action observed (Figure 4). Both wrists almost moved repeatedly with a dorsiflexion greater than 20° and maximum values of 54.2° (right) and 55.3° (left). The left wrist exhibited a large dorsiflexion posture with evenly distributed ulnar and radial deviation, whereas the right wrist exhibited an obvious ulnar deviation. The maximum movement range for both wrists during the dough rod operation was similar (Figure 5). The left and right wrist dorsiflexion was in an unsafe range. During the operation, the rolling pin is held in the right hand and force is applied using the left hand to roll the dough, causing the left wrist to reach its movement limit. The alterations in wrist movement and the maximum movement range during the dough rounding are shown in Figure 6 and Figure 7, respectively. During the operations, the movements of both wrists were relatively consistent, and the maximum radial deviation of the left (19.4°) and right wrist (16.8°) reached the limit. As shown in the figure, the radial deviations of both wrists were significant at approximately 5 s and then returned to ulnar deviation with slight up and down movement.

Wrist movement measurement indicated that movement repetitions during the 3 operations far exceeded safety recommendations, and the movement ranges of both wrists exceeded the safety limits suggested in previous studies. Among the 3 operations, the dough rounding operation was associated with a relatively small force exertion and conformed to the Principles of Motion Economy, according to which the 2 hands should begin and complete their motion at the same time, and arm movements should be symmetrical, simultaneous, and in opposite directions [31]. Thus, dough rounding is a less harmful operation than the others. This may explain the results of the logistic regression, which indicated that dough rounding was not a risk factor for upper limb disorders in these bakery workers.

Several limitations of this study should be highlighted. The NMQ survey is limited in that only 81 respondents in Northern Taiwan were recruited. The sample is relatively small compared to the population approximately 50,000 workers on the payroll in Taiwan. This limitation is because these bakery stores are frequently small and fragmental, even though 23 bakeries in Northern Taiwan were investigated in this study. The wrist posture measurement was performed only by 3 participants, and a larger sample should be recruited and examined in further validation. The results of this case analysis can serve as a pilot study to arouse greater attention in those who perform the bakery work every day.

## 5. Conclusions

This study focused on exploring musculoskeletal disorders symptoms in Taiwanese bakery workers. The study was divided into 2 parts. In the first part, the NMQ and logistic regression were used to analyze the prevalence of the disorders symptoms and the risk factors that may cause discomfort or injury to various body parts. In the second part, the dough processing operations were simulated, and the movements of left and right wrists were recorded during 3 common dough operations. The results revealed that the prevalence of overall discomfort among bakers within a year was 93.0%, with the prevalence in the hands/wrists, shoulders, and lower back being the highest. Dough operation simulation revealed that during the 3 types of operations, the maximum ulnar deviation/dorsiflexion of the right wrist in the workers was close to the movement limits defined in the literature, and the same phenomenon was observed for the left ulnar deviation. In summary, manual dough operations are a major hazard for bakery workers, and further improvement and redesign strategies are required.

## Figures and Tables

**Table 1:** Prevalence of musculoskeletal discomfort in various body parts between bakery workers and workers in other industries.

Body Parts	Entire Working Population (%)	Hospitality and Food Industry (%)	Recreation Industry (%)	Other Service Industries (%)	This Study (%) *
Neck	32.3	28.7	30.1	32.9	32.5
Shoulders	41.3	42.0	38.6	40.9	45.8/50.6
Upper back	22.3	23.4	20.8	23.8	32.5
Elbows	20.5	27.9	17.0	24.3	19.3/28.9
Lower back/waist	31.0	35.0	29.2	30.7	48.2
Hands/wrists	26.5	36.4	28.1	30.5	51.8/66.3
Buttocks/thighs	11.8	13.5	14.4	12.7	13.3/12.0
Knees	16.9	19.2	15.2	20.4	20.5/21.7
Legs/ankles	14.6	17.8	15.3	15.5	15.7/16.9

Source: Institute of Labor and Occupational Safety and Health (ILOSH) [24] and the present study; * Data in X/Y means left/right.

**Table 2:** Risk factors significantly associated with musculoskeletal disorders.

Body Parts (Prevalence %)	Risk Factors	Category	*n*	OR	95% CI
Right hand/wrist (66.3%)	Rolling pin use	Seldom	20	1.00	-
		Occasionally and more	61	4.30 *	1.36–13.61
	Dough kneading	Seldom	32	1.00	-
		Occasionally and more	49	4.23 *	1.40–12.81
	Stature	<170 cm	35	1.00	-
		≥170 cm	46	3.73 *	1.25–11.19
Left hand/wrist (51.8%)	Rolling pin use	Seldom	20	1.00	-
		Occasionally and more	61	4.57 *	1.33–15.73
	Dough kneading	Seldom	32	1.00	-
		Occasionally and more	49	6.13 ***	2.05–18.32
	Stature	<170 cm	35	1.00	-
		≥170 cm	46	6.82 **	2.16–21.52
Right shoulder (50.6%)	Rolling pin use	Seldom	20	1.00	-
		Occasionally and more	61	5.29 **	1.76–15.89
	Lifting objects	Seldom	33	1.00	-
		Occasionally and more	48	14.50 **	3.19–29.83
	Number of times pastries are removed out of the oven per day	<20 trays	38	1.00	-
		≥20 trays	43	9.04 ***	3.31–26.91
	Working hours per day	<9 h	40	1.00	-
		≥9 h	41	5.08 ***	1.83–14.70
	Job tenure	<10 y	56	1.00	-
		≥10 y	25	7.56 ***	2.20–25.92
	Age	<30 y	49	1.00	-
		≥30 y	32	5.25 ***	1.86–14.80
Lower back /waist (48.2%)	Rolling pin use	Seldom	20	1.00	-
		Occasionally and more	61	2.54 *	0.98–6.54
	Lifting objects	Seldom	33	1.00	-
		Occasionally and more	48	4.69 ***	1.81–12.14
	Number of times pastries are removed out of the oven per day	<20 trays	38	1.00	-
		≥20 trays	43	4.55 ***	1.77–11.66
	Age	<30 y	49	1.00	-
		≥30 y	32	4.04 *	1.30–12.53
Left shoulder (45.8%)	Rolling pin use	Seldom	20	1.00	-
		Occasionally and more	61	3.88 **	1.37–10.98
	Lifting objects	Seldom	33	1.00	-
		Occasionally and more	48	9.75 ***	3.19–29.83
	Number of times pastries are removed out of the oven per day	<20 trays	38	1.00	-
		≥20 trays	43	5.54 **	1.99–15.42
	Working hours per day	<9 h	40	1.00	-
		≥9 h	41	4.24 *	1.56–11.52
	Job tenure	<10 y	56	1.00	-
		≥10 y	25	4.85 **	1.60–14.77
	Age	<30 y	49	1.00	-
		≥30 y	32	4.24 **	1.55–11.60

* *p* < 0.05, ** *p* < 0.01, *** *p* < 0.001. OR—odds ratio; CI—confidence interval.
